# Chronic modafinil therapy ameliorates depressive-like behavior, spatial memory and hippocampal plasticity impairments, and sleep-wake changes in a surgical mouse model of menopause

**DOI:** 10.1038/s41398-021-01229-6

**Published:** 2021-02-08

**Authors:** Yu-Dong Yan, Yu-Qing Chen, Chen-Yao Wang, Chen-Bo Ye, Zhen-Zhen Hu, Thomas Behnisch, Zhi-Li Huang, Su-Rong Yang

**Affiliations:** 1 Department of Pharmacology, School of Basic Medical Sciences; State Key Laboratory of Medical Neurobiology and MOE Frontiers Center for Brain Science, Fudan University, Shanghai, 200032 China; 2 Institutes of Brain Science, State Key Laboratory of Medical Neurobiology and MOE Frontiers Center for Brain Science, Fudan University, Shanghai, 200032 China; 3 Department of Ophthalmology, Zhongshan Hospital, Fudan University, Shanghai, 200032 China

**Keywords:** Learning and memory, Molecular neuroscience, Psychiatric disorders, Pharmacology

## Abstract

Depression, cognitive deficits, and sleep disturbances are common and often severe in menopausal women. Hormone replacement cannot effectively alleviate these symptoms and sometimes elicits life-threatening adverse reactions. Exploring effective therapies to target psychological problems is urgently needed. In this work, we developed a mouse model of menopause by bilateral ovariectomies (OVXs) and investigated whether menopausal mental symptoms can be ameliorated by psychostimulant modafinil (MOD) as well as explored the underlying mechanisms. At ~3 weeks after OVXs, mice got daily intraperitoneal administrations of MOD at the beginning of the active phase. Several behavioral tests and electroencephalogram (EEG) recordings were conducted. Electrophysiological and immunohistochemical experiments were carried out to evaluate the synaptic plasticity and neurogenesis, respectively. We found that chronic MOD administration in OVX mice significantly decreased immobility time. The spatial memory performance of OVX mice improved significantly in response to MOD administration in the Morris water-maze test. The OVX mice were characterized by an attenuation of hippocampal synaptic transmission and synaptic long-term potentiation and had fewer 5-ethynyl-2′-deoxyuridine-labeled cells in the dentate gyrus, which were restored after MOD administration. Antagonists of dopamine D_1_ and D_2_ receptors and GABA_A_ receptor agonists were involved in MOD-exerted anti-depressant actions and augments of hippocampal neurogenesis in OVX mice. Moreover, night-dosed MOD therapy significantly promoted the night-time delta-band EEG power during wakefulness and the day-time rapid eye movement sleep amount, which were significantly reduced by OVXs. Collectively, these findings suggest that MOD is a promising therapeutic candidate for menopausal women.

## Introduction

With the increase in life expectancy, women spend more than one-third of their lives in menopause^1^. Additionally, bilateral ovariectomies (OVXs) performed for the treatment of ovarian pathologies or as a prophylactic procedure for reducing the risk of breast and ovarian cancer are a leading cause of ovarian hormonal deficiencies in pre-menopausal women. Surgery-induced menopausal symptoms are often more sudden in onset, more frequent, and more severe compared to those from natural age-induced menopause^2^. The current hormone replacement therapy is inadequate, with insufficient effectiveness, several limitations, and adverse reactions^3^.

During the menopausal transition, the risk of a major depressive disorder is 2–4 times greater than at the premenopausal stage^4^. Depression greatly and negatively affects daily functioning, quality of life, and imposes a severe burden on inflicted individuals and their families^5^. Additionally, a wide range of cognitive problems, including forgetfulness and decreases in attention, executive function, and spatial working memory, are common in women transitioning through menopause^6^. Sleep disturbances that represent another common and recurring condition have been self-reported in 40–60% of perimenopausal women^7^.

The pathophysiologic changes associated with menopause in the brain are varied and complicated. Some evidence has shown that OVX rats have neuron loss in some brain areas, including the hippocampus, hypothalamus, and amygdala, which are crucially related to memory and emotion processing and sleep-wake regulation. Furthermore, the OVX rats show activation of autophagy, an imbalance between excitatory and inhibitory neurotransmission, and glutamic synaptic impairments^8–11^.

Modafinil (MOD), a psychostimulant, has been approved for the treatment of excessive daytime sleepiness in narcolepsy and obstructive sleep apnea. Compared to traditional stimulants, such as amphetamine, MOD is well-tolerated, shows fewer or no adverse effects, and has a comparatively low potential for abuse^12^. Moreover, MOD has been proposed to improve cognitive performance in normal rats^13–15^ or mice with sleep deprivation^16^, healthy human volunteers^17^, and patients with schizophrenia or depression^18,19^. Furthermore, an effective augmentation strategy for ameliorating depressive episodes in patients with depression has been revealed, indicated by significant improvements in overall depression scores and remission rates when MOD is administered combined with an antidepressant^20^. On the cellular level, MOD administration affects the synaptic transmission, increases precursor cell proliferation, and suppresses excessive autophagy and apoptosis in the hippocampus^15,16,21^.

Given the tremendously detrimental impacts of neuropsychological menopausal symptoms, it is imperative to discover and develop treatments that are safe and effective for dealing with an array of symptoms women experience as they transition to menopause. Whether MOD can simultaneously improve the menopausal symptoms remains to be elucidated. In this study, we utilized a mouse model of bilateral OVXs that presented menopausal-like symptoms^22^ to investigate neurobehavioral functions after daily repeated MOD administration and the potential mechanisms. Considering that the half-life of MOD is ~15 h^23^ and that it exerts multimodal actions, we hypothesized that chronic MOD therapy would elicit long-term improvements in OVX-mediated symptoms in mice.

## Materials and methods

### Animals

Female C57BL/6 mice were at eight to ten weeks of age at the start of each experiment. They were housed four to five per cage with access to food and water *ad libitum* at a constant temperature (22–24 °C) and humidity (40–60%), with a 12-h light (inactive)/dark (active) cycle (lights on at 07:00). The animals were randomly allocated in each experiment. All experimental protocols performed were approved by the Committee on the Ethics of Animal Experiments in the School of Basic Medical Sciences at Fudan University, with license identification number 20190221-014. All efforts were made to minimize animal suffering and discomfort and the number of animals used.

### Experimental design and pharmacological treatments

Mice were received bilateral OVXs under anesthesia with sodium pentobarbital at 50 mg/kg (intraperitoneal). For sham operations, fat tissue surrounding the ovaries was excised instead. Following OVX and sham surgeries, a soy-free diet was given to exclude the impact of soy phytoestrogens^24^. MOD (Sigma-Aldrich) was dissolved in sterile saline containing 10% dimethyl sulfoxide and 2% (w/v) Cremophor immediately before use^25^. The target doses reflect concentrations commonly clinically used for humans of about 100–400 mg/d^12,23^. MOD or vehicle began at 23 days post-surgery and was intraperitoneally administered at 19:00 (light offset) each day until experiments were completed. The concentration of estradiol (E_2_) in blood was detected using an enzyme-linked immunosorbent assay (ELISA) kit (Servicebio Technology, China)^26^.

To explore mechanisms of MOD treatment on behavioral changes in OVX mice, separate pharmacologic experiments were conducted. SCH23390 (SCH, 30 µg/kg, Sigma–Aldrich, USA), a selective dopamine D_1_ receptor antagonist, raclopride (Rac, 2 mg/kg, Sigma–Aldrich, USA), a selective dopamine D_2_ receptor antagonist, and a selective GABA_A_ receptor agonist diazepam (DZP, 20 µg/kg, King York, China) was dissolved in sterile saline, respectively. Either one or a mixture of SCH and Rac was administered intraperitoneally to OVX mice 30 min before each MOD (45 mg/kg) administration.

### Behavioral testing

Before each test, mice were habituated in the testing room for an hour. All of the behavioral tests except the locomotor tests were carried out during the inactive phase. All the behavioral tests were carried out and analyzed by investigators blinded to the group information.

#### Open field test (OFT)

The OFT apparatus was made of white opaque plastics (50 cm × 50 cm × 50 cm) and was softly illuminated. Each mouse was placed gently on the central area (25 cm × 25 cm) and was allowed to explore for 10 min. The movement was analyzed by the EthoVision tracking system (Noldus, Netherlands)^27^.

#### Elevated plus-maze test (EPT)

The apparatus consisted of two open arms (35 cm × 6 cm each), two closed arms (50 cm × 8 cm) with 20-cm-high transparent walls, and a central platform. The entire maze was elevated 70 cm above the floor. Each mouse was placed on the central platform facing one open arm. The movement was recorded and analyzed during a 5-min period by ANY-Maze (Stoelting, USA)^28^.

#### Locomotor activity

Locomotor activities of an individual mouse were detected for 24 h by an infrared sensor (Biotex, Japan) placed above the floor of a recording cage.

#### Tail suspension test (TST)

Each mouse was suspended by its tail with tape at about 50 cm above the floor. The duration of immobility over the last 4 min in a 6-min session was calculated^29^.

#### Forced swimming test (FST)

Each mouse was placed into a plastic cylinder filled with water at 22–26 °C. Floating and slight movements made to keep the mouse’s head above water were considered immobility. The duration of immobility during the final 4 min of a 6-min session was calculated^30^.

#### Morris water maze test (MWT)

The spatial performance was evaluated in a task using a submerged platform that remained at the same location for all trials. Each mouse was allowed to swim for a maximum of 60 s and to stay on the platform for 10 s whether the mouse found the platform by itself or with the experimenter’s guidance. There were four trials for each mouse per day, which continued for seven days. In the single test trial, the platform was removed, and each mouse was allowed to swim for 60 s. All swimming tracks were recorded and analyzed by the EthoVision tracking system (Noldus, Netherlands)^31^.

### Electrophysiology

For hippocampal slice preparation, briefly, after ether anesthesia, the mouse’s brain was removed rapidly and immersed in oxygenated ice-cold modified Gey’s solution. The dissected hemispheres were fixed using glue on the slicing platform of a vibratome (Vibratome 3000, St. Louis, MO, USA). Transverse hippocampal slices (350 μm) were transferred to a custom-made interface recording chamber. The incubation under constant perfusion with oxygenated artificial cerebrospinal fluid (ACSF) at 32 °C lasted at least 1 h. Field excitatory postsynaptic potentials (fEPSPs) were induced by stimulation of the Schaffer collateral fibers with biphasic rectangular current pulses (100 μs/polarity) in a range of 15–30 μA through stainless steel electrodes (A-M Systems, Inc., Sequim, WA, USA). Data were recorded and acquired from CA1 stratum radiatum using a differential amplifier (EXT-20F; npi electronic GmbH, Tamm, Germany) with a 3 kHz low-pass and 0.1 Hz high-pass filter. The recorded field potentials were digitized at a frequency of 10 kHz by a CED 1400 plus AD/DA converter (Cambridge Electronic Design, Cambridge, UK).

The stimulation electrodes were placed in the stratum radiatum area of the hippocampal CA1 region. A recording electrode was placed in-between to record fEPSPs evoked by extracellular stimulation of Schaffer collaterals by S1 input. The stimulation strength was adjusted to generate 40% of the maximum fEPSP-slope values through high current testing. After a stable baseline recording testing, paired-pulse facilitation (PPF) was obtained at 10, 30, 50, 70, 100, 150, and 200 ms inter-stimulus intervals. The input–output curve was plotted as the fEPSP slope against stimulus intensity. Long-term potentiation (LTP) was induced by two trains of theta burst stimulation (TBS) with an interval of 30 s. Each train contained 10 bursts at an interval of 200 ms (5 Hz), and each burst included 5 pulses at 100 Hz. A set of four fEPSPs at an inter-stimulus interval of 10 s were evoked and averaged every 5 min (0.033 Hz). The delivery of presynaptic stimulation and data acquisition and analysis were carried out using PWIN software (IfN, Magdeburg, Germany)^32^. The investigator was blinded to the group information during the experiment and data processing.

### Neurogenesis assay

A thymidine analog, 5-ethynyl-2′-deoxyuridine (EdU) (50 mg/kg, RiboBio, C00054, China), was intraperitoneally injected 30 min after the first and fifth day of MOD administration to detect DNA synthesis in proliferating cells^33^. Double immunostainings of doublecortin (DCX), a microtubule-associated protein expressed by immature neurons^34^, and EdU were performed (rabbit polyclonal anti-DCX antibody: 1:1.000, Abcam, ab18723, UK). The total number of EdU^+^ cells in the granule cell layer of the dentate gyrus (DG) (−1.22 to −2.30 mm from Bregma) was counted in a blinded manner to the group information^21^. Each labeled cell within the target area was counted every four slices.

### Polygraphic recordings and vigilance state analysis

At approximately two weeks after OVXs and sham operations, mice were implanted with the electroencephalogram (EEG) and electromyogram (EMG) electrodes for polysomnographic recordings. Cortical EEG and EMG signals were amplified, filtered (EEG, 0.5–30 Hz; EMG, 20–200 Hz), digitized at a sampling rate of 128 Hz, and recorded using VitalRecorder (Kissei Comtec, Japan). Once complete, polygraphic recordings were automatically scored offline into 10-s epochs that were categorized as either waking, non-rapid eye movement (NREM) sleep, or rapid eye movement (REM) sleep using SleepSign according to standard criteria. Defined sleep-wake stages were examined visually and corrected if necessary by investigators blinded to the group information. A bout was defined as an episode without interruptions of any other vigilance stage. For EEG spectral power analysis, fast Fourier transformations (FFTs) were performed for all consecutive 10 s epochs. Then the FFT data of each vigilance stage (wakefulness, NREM, REM) in the frequency range 0–24.5 Hz (frequency resolution of 0.5 Hz) during the 12-h inactive and active phase were averaged, respectively. The spectral power density at a specific frequency was expressed as the percentage of total FFT data of the frequency range 0–24.5 Hz of each animal in order to minimize individual variations^35,36^.

### Statistical analysis

The sums of sleep/wakefulness and sleep architecture parameters, as well as the data from behavioral experiments, MWT probe trials, and neurogenesis assays were compared among multiple groups using one-way analysis of variance (ANOVA). Unpaired *t*-tests were used when the data between two groups were compared. The hourly durations of sleep/wakefulness, landing rates, latencies, and swimming speed in the MWT as well as locomotor analyses were assessed using two-way repeated ANOVA. Tukey’s post hoc comparisons were performed when appropriate. Data from the electrophysiology experiment were normalized to baseline values, presented in percentages, and then compared via Mann–Whitney *U*-tests using SPSS software. Values are presented as mean ± standard error of the mean (SEM). In all cases, a *p* < 0.05 was considered to be statistically significant.

## Results

### Effects of MOD on uterus weights and E2 levels following OVXs

**Fig. 1:**
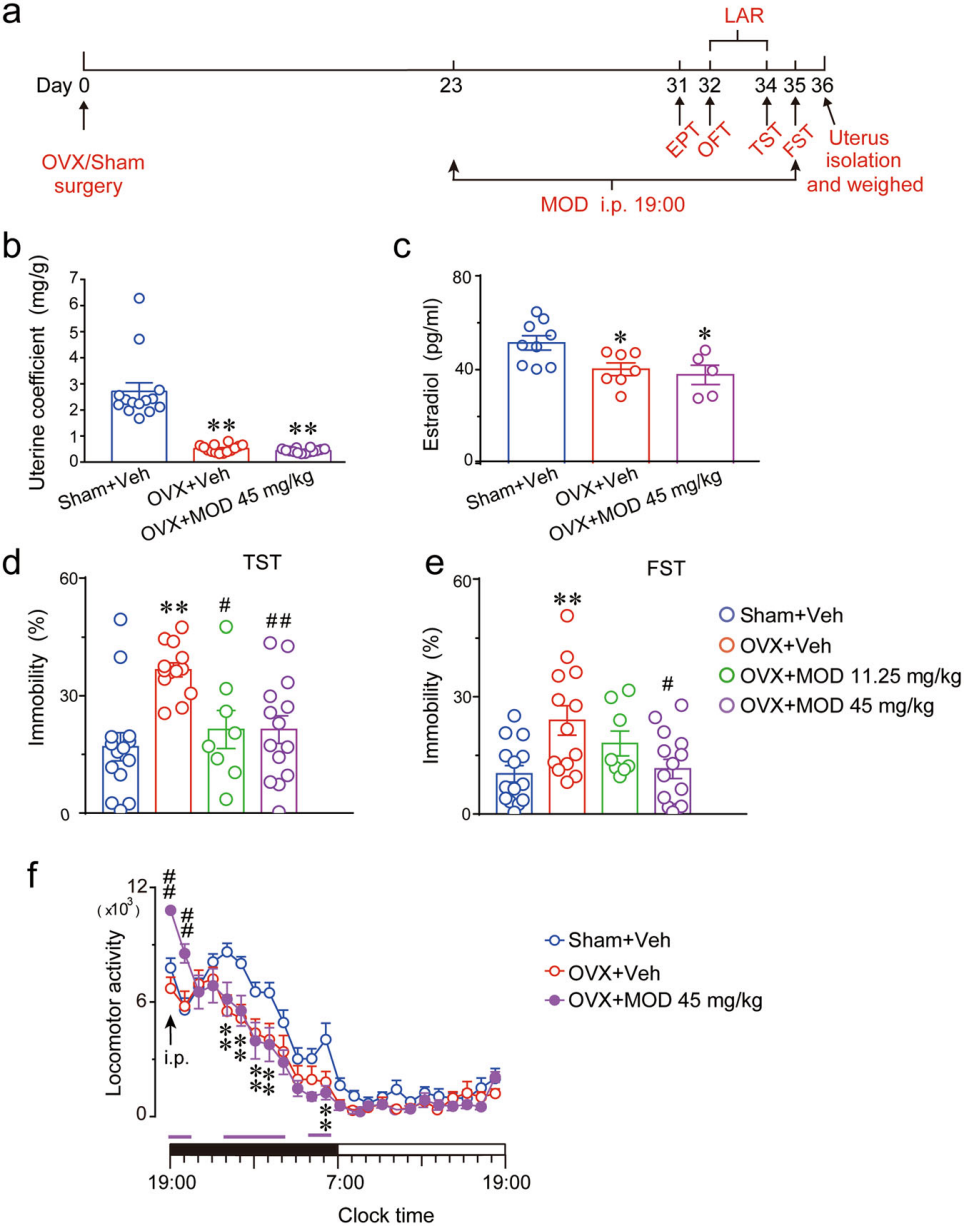
Modafinil reverses depressive-like behaviors but has no effects on the reduction of uterus weight and estradiol concentration in ovariectomized mice. **a** Behavioral tests were conducted during the modafinil (MOD) administration. On the day after the last MOD injection, the uterus of each mouse was separated and weighed. **b** The uterus coefficient was calculated as ratio of the uterine weight and body weight of each mouse at the end of the experimental sequences after 13-day MOD administration. *n* = 14 per group. **c** The concentration of estradiol in serum determined by enzyme-linked immunosorbent assay in each group. Sham+Vehicle (Veh): *n* = 9; OVX+Veh: *n* = 7; OVX+MOD 45 mg/kg: *n* = 5. **d**, **e** Percentage of immobility time in the TST (**d**) and FST (**e**) carried out at 11 days and 12 days, respectively, after MOD injections. Sham+Veh, OVX+MOD 45 mg/kg: *n* = 14 per group, OVX+Veh: *n* = 13, OVX+MOD 11.25 mg/kg: *n* = 8. *p < 0.05, **p < 0.01 compared with the Sham+Veh group; ^#^*p* < 0.05, ^##^*p* < 0.01 compared with OVX+Veh group in (**b**–**e**). **f** Time-course changes of the locomotion activity after 10 days of MOD treatment. **p < 0.01 OVX+Veh group compared with the Sham+Veh group; horizontal pink bars under the curve indicate statistical differences (*p* < 0.05) of the OVX+MOD group versus Sham+Veh group; ^##^*p* < 0.01 OVX+MOD group compared with OVX+Veh group. Sham+Veh, OVX+MOD 45 mg/kg: *n* = 15 per group, OVX+Veh: *n* = 13. The horizontal filled and open bars on the *x*-axes indicate the 12-h active phase and 12-h inactive phase, respectively. EPT elevated plus-maze test, OFT open field test, LAR locomotor activity recording, TST tail suspension test, FST forced swimming test.

Figure 1a shows the protocol for this part of the experiment. We found that the OVX mice showed substantial atrophy of the uterus [*F*(2,39) = 43.79, *p* < 0.0001] and a significant decrease in serum E_2_ concentration [*F*(2,18) = 5.307, *p* = 0.0154] compared with the sham group, indicating that OVXs were successful. Starting on the 23rd day after OVXs, MOD administration was begun. Chronic MOD treatments for 13 days did not increase the OVX-mediated decrease in weights of the uteri and levels of E_2_ in the serum of mice (Fig. 1b, c), indicating that MOD had no estrogen-like actions.

### Depressive-like behavior induced by OVXs is alleviated by MOD administration

First, anxiety-like behavior was determined. There were no differences among the different groups in terms of the distance traveled or time spent in the center of the OFT, or the percentage of distance in the open arms or entries into the open arms in the EPT (Fig. S1). Then, the immobility durations of mice were calculated to assess behavioral despair. OVX mice showed a longer immobility time compared to sham-operated mice, whereas MOD at 45 mg/kg ameliorated OVX-induced increases in immobility in both the TST [*F*(3, 45) = 6.766, *p* = 0.0007] and FST [*F*(3, 45) = 5.045, *p* = 0.0142] (Fig. 1d, e). These results indicated that chronic MOD treatment alleviated the depressive-like behavior of mice induced by OVXs.

Locomotor activities were detected by infrared sensors. A two-way repeated ANOVA revealed a significant difference in the locomotion among the three groups [*F*(2, 40) = 7.062, *p* = 0.0024]. During the active phase, OVX mice exhibited significantly decreased locomotion compared to the sham group. Moreover, MOD-induced increases in locomotor activity lasted for approximately 2 h following MOD administration on the 10th day of drug treatment, after which MOD-treated mice exhibited decreased locomotion that was similar to OVX mice (Fig. 1f).

### MOD ameliorates OVX-induced spatial memory deficits

**Fig. 2:**
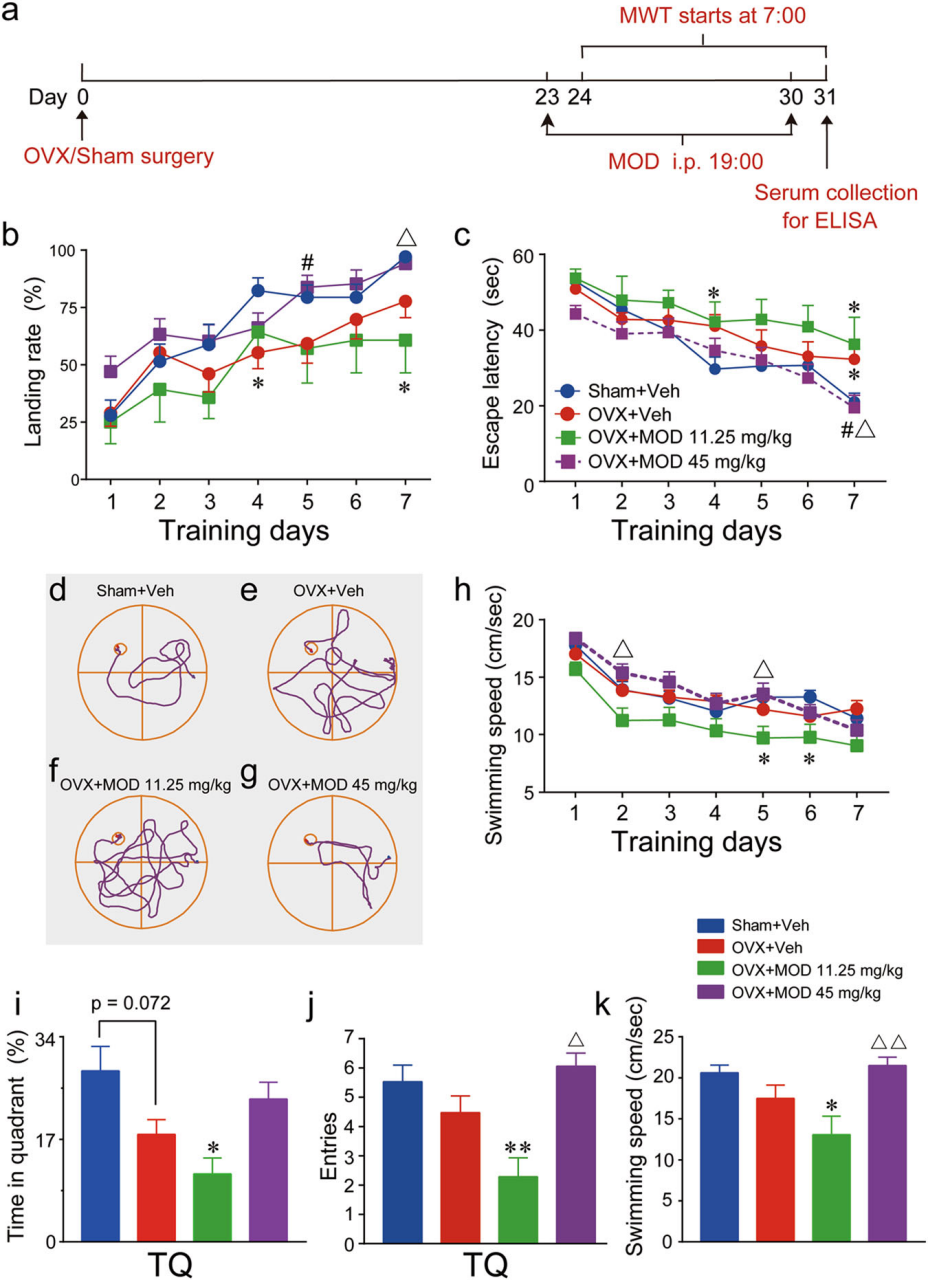
Modafinil ameliorates ovariectomy-induced spatial memory deficits in the Morris water-maze test. **a** The Morris water-maze test (MWT) was conducted on the next morning after modafinil (MOD) was administered the night before. Serum was collected for estradiol evaluation using an enzyme-linked immunosorbent assay (ELISA). **b** The platform-landing rate, denoting the percentage of trials in which the submerged platform was found without guidance among the four trials for each mouse per day, of each group during the seven training days. **c** The average latencies of mice in different groups to find the hidden platform on each day of the seven training days. **d**–**g** Typical swimming traces of the mouse in the Sham+Vehicle (Veh) (**d**), OVX+Veh (**e**), OVX+MOD 11.25 mg/kg (**f**), and OVX+MOD 45 mg/kg (**g**) groups recorded on the seventh training day. **h** Average swimming speed of mice in different groups during the seven training days [group: *F*(3, 56) = 4.203, *p* = 0.0094]. **i**–**k** The memory of the platform location was assessed in a probe trial in the absence of the platform on the eighth day morning, while MOD was intraperitoneally injected at 19:00 on the seventh day of MOD treatment. The time spent in the target quadrant (TQ) [*F*(3, 56) = 3.883, *p* = 0.0136] (**i**), entries into the TQ [*F*(3, 56) =5.504, *p* = 0.0022] (**j**), and mean swimming speed during the 1-min probe test [*F*(3, 56) = 4.962, *p* = 0.0040] (**k**). *p < 0.05, **p < 0.01 compared with the Sham+Veh group; ^#^*p* < 0.05 compared with OVX+Veh group; ▵*p* < 0.05, ▵▵*p* < 0.01 compared with OVX+MOD 11.25 mg/kg group. Sham+Veh, OVX+MOD 45 mg/kg: *n* = 17 per group, OVX+Veh: *n* = 19, OVX+MOD 11.25 mg/kg: *n* = 7.

Spatial memory in rodents has most often been assessed via a fixed hidden platform in the MWT, in which rodents have to remember the location of the platform across trials. Figure 2a shows the protocol of the MWT. Considering that successful discovery of the platform by each mouse is learned during training, besides escape latencies, we also analyzed the percentage of trials in which mice found the hidden platform without guidance among the four groups during the entire seven-day acquisition process in the MWT. With each day of training, the mice in different groups showed significantly improved spatial performance on experimental days, as demonstrated by increased platform-landing rates [time: *F*(6, 336) = 23.38, *p* < 0.0001] and decreased escape latencies [time: *F*(6, 336) = 25.7, *p* < 0.0001]. Moreover, two-way ANOVAs revealed significant effects of treatments on the platform-landing rates [group: *F*(3, 56) = 5.065, *p* = 0.0036] and escape latencies to locate the hidden platform [group: *F*(3, 56) = 3.633, *p* = 0.0182] during the whole seven-day training process. The percentage of trials in which OVX mice successfully found the platform was significantly lower than that of the sham group on the fourth training day, whereas OVX mice receiving MOD treatments (45 mg/kg) exhibited a higher percentage of successful trials on the fifth training day (Fig. 2b). OVX mice spent more time locating the invisible platform compared to the sham-operated mice on the fourth and seventh training days. MOD-treated OVX mice demonstrated better performance with less time to locate the hidden platform compared to vehicle-treated OVX mice on the seventh training day (Fig. 2c). The typical swimming traces on the seventh training day showed that the OVX mice effectively searched for and located the platform, which was improved by higher-dose MOD treatment (Fig. 2d–g). Among the sham, OVX, and higher-dose MOD treatment groups, no significant differences in swimming speed were found (Fig. 2h). Thus, swimming speed was not a possible factor contributing to the OVX-induced deficits and higher-dose MOD-induced improvements in the spatial acquisition capabilities of mice in the WMT.

To determine the effects of chronic MOD treatment on retrieval of spatial memory, mice were given a 60-s probe trial in the absence of the platform on the next morning after the eighth MOD injection at night. Compared with the sham mice, the OVX mice tended to spend less time in the target quadrant and have fewer entries into the target quadrant, which could be alleviated to some extent by higher-dose MOD therapy, but these differences were not statistically significant (Fig. 2i–k).

### MOD ameliorates OVX-induced deficits in hippocampal neural plasticity

**Fig. 3:**
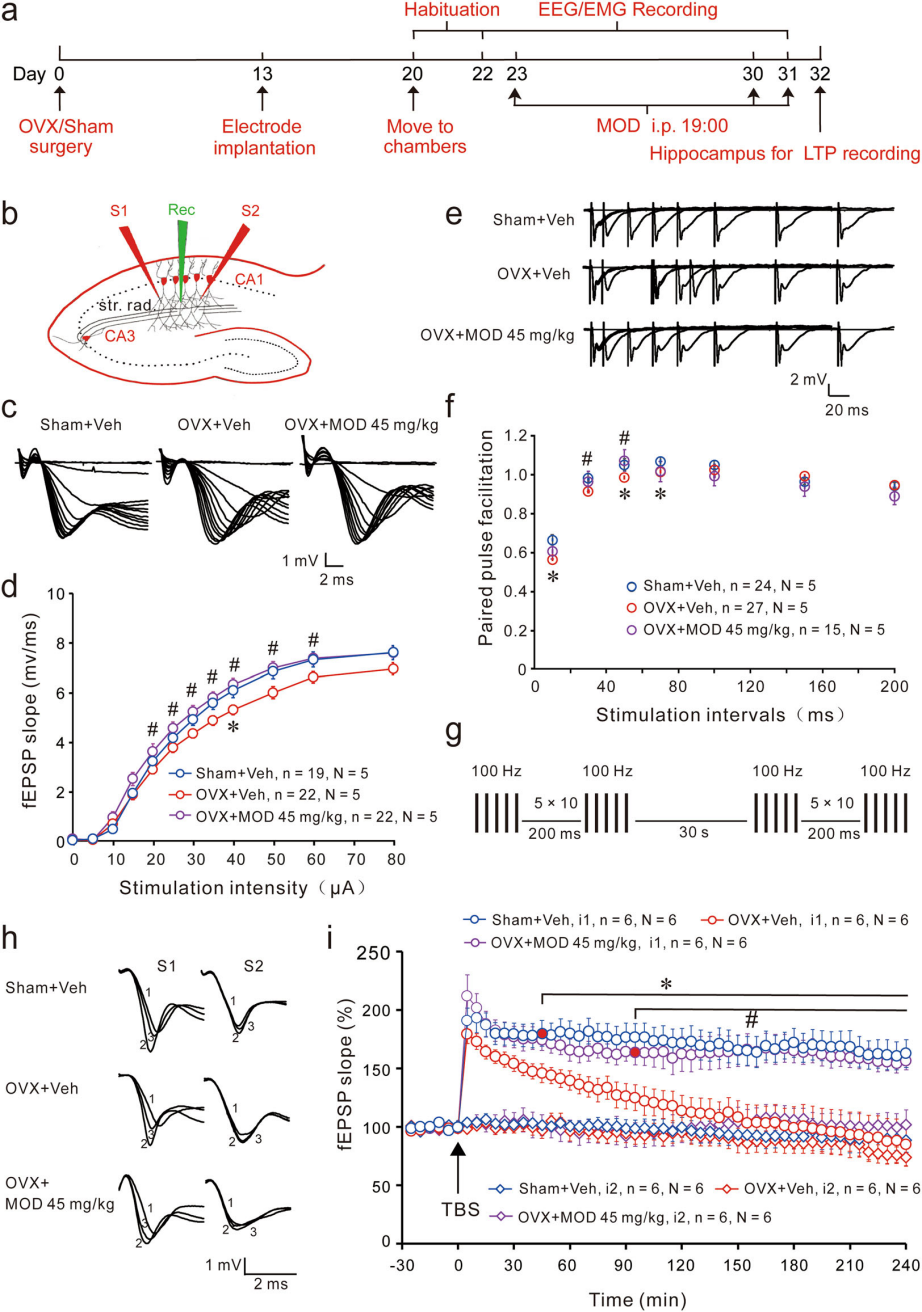
Modafinil rescues ovariectomy-induced impairments of hippocampal basal transmission and long-term potentiation in hippocampal slices ex vivo. **a** At the end of sleep recordings, the hippocampus of each animal was separated for electrophysiological experiments. **b** Diagram of a hippocampal slice showing the placement of electrodes. The stimulation electrodes (red, S1, S2) were placed in the stratum radiatum (str. rad.) area of the hippocampal CA1 region. A recording electrode (green, Rec) was placed in between to record field excitatory postsynaptic potentials (fEPSPs) evoked by extracellular stimulation of Schaffer collaterals by the S1 input. The S2 input was used as a control input to monitor baseline stability. **c** Representative traces of fEPSPs obtained after a set of consecutive stimuli. **d** The input-output relationship of basal synaptic transmission was obtained by plotting the slope of fEPSPs against stimulation intensity in different groups of mice. **e** Sample fEPSP traces of paired-pulse facilitation (PPF, ratios of the second fEPSP and the first fEPSP obtained at different interstimulus intervals). **f** PPF was obtained in different groups of mice. *p < 0.05 versus Sham+Vehicle (Veh) group; ^#^*p* < 0.05 versus OVX+Veh group assessed by Mann–Whitney *U* tests in (**d**) and (**f**). **g** Schematic representation of the stimulation protocols used for the induction of long-term potentiation (LTP). Two trains of theta-burst stimulation (TBS) with a 30-s inter-train interval were delivered to induce LTP. **h** Sample traces of fEPSPs at 0, 5, and 180 min (marked as 1, 2, and 3, respectively). **i** Time courses of normalized initial slope measurements of fEPSPs. Every data point represented the averaged data of four consecutive sweeps with an interval of 10 s. LTP was evoked by TBS indicated by black arrowheads. *p < 0.05 versus Sham+Veh group; ^#^*p* < 0.05 versus OVX+Veh group. The black horizontal bars cover all the significantly different time points. *N* indicates the numbers of animals, while *n* indicates the numbers of slices. The non-tetanized baseline recording by input S2 remained stable in the different groups.

The hippocampus, a part of the limbic system, plays a crucial role in the consolidation of memory and is implicated in mood disorders^37^. To determine the underlying cellular mechanisms of the improvements of MOD therapy on cognitive deficits and depression in OVX mice, we next examined synaptic physiology and neurogenesis in the hippocampal formation after sleep recording (Fig. 3a). The input–output relationship (I/O ratio) is a sensitive measure of synaptic connectivity, which reflects the number of active axons required to elicit a given postsynaptic response. The I/O ratio in the OVX group was lower than that in the sham group, which reached a significant difference at 40 µA of stimulation strength. Compared with the vehicle-treated OVX group, the MOD-treated group showed a significant increase in basal synaptic transmission at Schaffer-collaterals to CA1 synapses at a range from 20 to 60 μA of stimulation intensities (Fig. 3b–d). To evaluate alterations of presynaptically mediated mechanisms of synaptic transmission, the PPFs of fEPSPs were analyzed. We found that PPF was decreased significantly in the OVX group at the stimulation intervals of 10, 50, and 70 ms. After MOD treatment, the OVX-induced PPF deficit was significantly improved at the stimulation intervals of 30 and 50 ms (Fig. 3e, f). Compared with the sham group, there were no significant differences in either fEPSPs in response to stimulation intensities or the PPF at any stimulation interval of the MOD group (Fig. 3d, f). These results suggest that OVXs induced impairments in synaptic transmission at CA3–CA1 synapses that were ameliorated by MOD therapy.

At 0, 5, and 180 min, the average fEPSP slopes in the OVX group were 98.6%, 179.4%, and 84.6%, respectively; in contrast, the average fEPSP slopes in the sham group were 99.3%, 190.7%, and 162.7%, respectively. From 45 min after LTP induction, there was a significant decline in LTP maintenance in the OVX group. At 0, 5, and 180 min, the average fEPSP slopes in the MOD-treated OVX group were 99.0%, 211.9%, and 156.1%, respectively. The LTP level in the MOD-treated OVX group was similar to that of the sham group (Fig. 3g–i). Our results indicate that MOD treatment completely restored the OVX-induced decline of LTP maintenance.

**Fig. 4:**
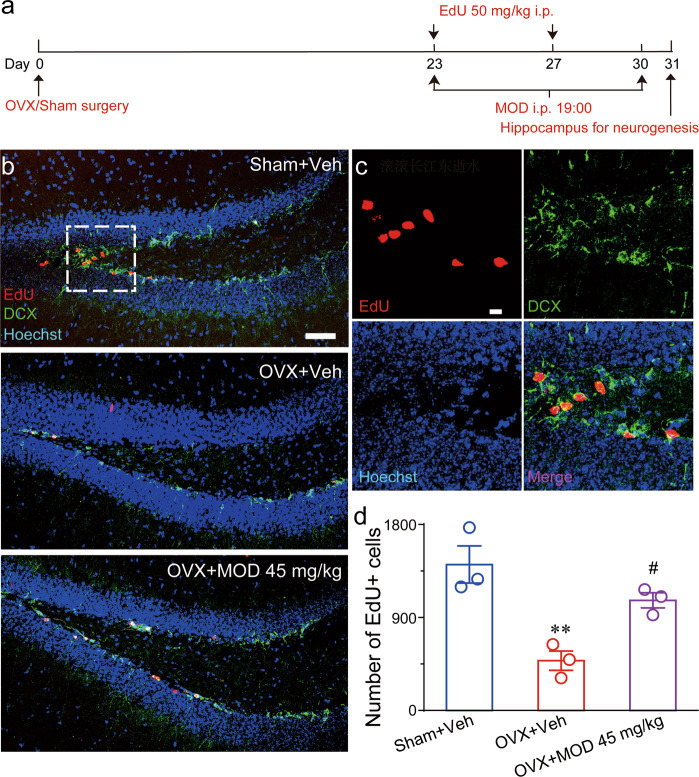
Modafinil ameliorates ovariectomy-induced reductions of newborn neurons in the hippocampal dentate gyrus. **a** Experimental design for neurogenesis staining. **b** Representative images of EdU/DCX immunostaining in the dentate gyrus in mice of each group. Scale bar: 50 μm. **c** Higher-magnification images of the area marked by the frame in (**b**). Scale bar: 20 μm. **d** Quantification of EdU+ cells generated during the eight-day modafinil (MOD) treatment [*F*(2,6) = 14.2, *p* = 0.0053]. *n* = 3 per group. **p < 0.01 compared with the Sham+Vehicle (Veh) group; ^#^*p* < 0.05 compared with ovariectomy (OVX)+Veh group. EdU 5-ethynyl-2′-deoxyuridine, DCX doublecortin.

Considering that the DG is a major source of afferent inputs into the hippocampus, DG-specific neurogenesis may have an important role in hippocampal functions^38^. To test the neurogenesis ability in OVX mice and the effect of MOD, the experiment scheme was designed as shown in Fig. 4a. We found that EdU-labeled neurons were mainly located in the DG of the hippocampus and co-expressed DCX (Fig. 4b, c). OVX mice had an approximately 65% lower number of EdU+ cells compared with sham mice; in contrast, long-term MOD treatment (45 mg/kg) in OVX mice significantly increased the number of newborn cells compared to vehicle treatment (Fig. 4d). These results indicate that MOD treatment ameliorated OVX-induced deficits in hippocampal neurogenesis.

### Dopamine D_1_/D_2_ and GABA_A_ receptors mediate the anti-depressant effects and neurogenesis-promotion of MOD in OVX mice

**Fig. 5:**
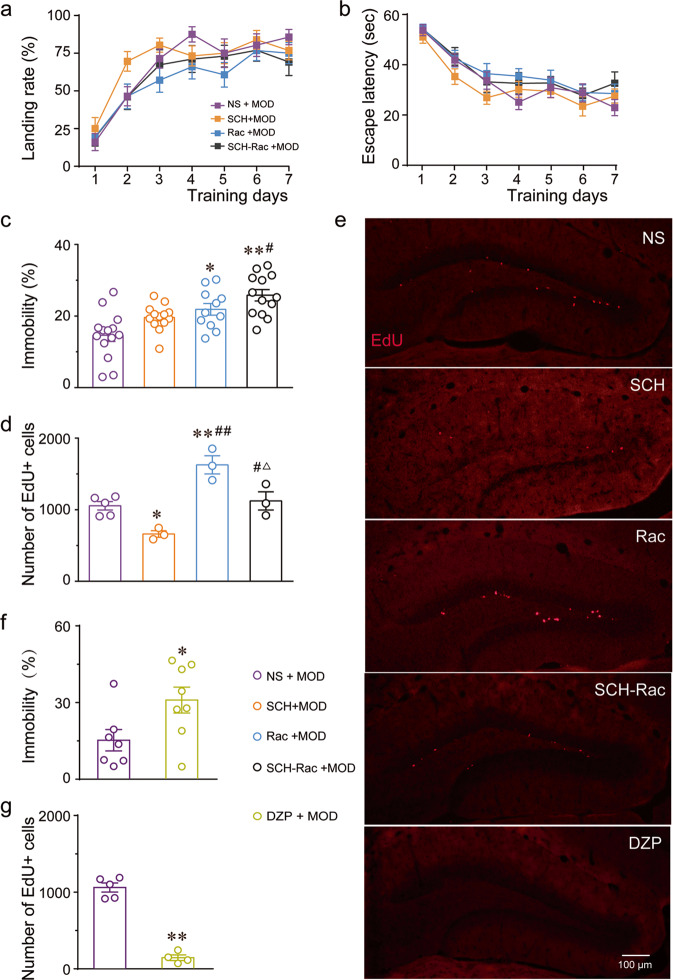
Dopamine and GABA_A_ receptors mediate MOD-produced anti-depressant effects and hippocampal neurogenesis-promotion in ovariectomized mice. **a–d** In ovariectomized mice treated with modafinil (MOD) at 45 mg/kg in the presence of saline (NS), a selective D_1_ receptor antagonist SCH23390 (SCH), a selective D_2_ receptor antagonist raclopride (Rac), and combined use of SCH and Rac, respectively, the landing rates (**a**) and escape latencies (**b**) locating the invisible platform during the seven training days in the Morris water-maze test (NS, SCH, Rac: *n* = 14 per group; SCH–Rac: *n* = 13), percentage of immobility time in the forced swimming test (FST) (**c**) (NS: *n* = 14; SCH, SCH–Rac: *n* = 13 per group; Rac: *n* = 11), and the numbers of newborn hippocampal neurons (**d**) (NS: *n* = 5; SCH, Rac, and SCH-Rac: *n* = 3 per group). **e** Representative images of EdU staining in the hippocampal dentate gyrus in each group of mice with ovariectomy (OVX). **f**, **g** In OVX mice treated with MOD in the presence of NS or a selective GABA_A_ receptor agonist diazepam (DZP), the percentage of immobility time in the FST (**f**) (NS: *n* = 7; DZP: *n* = 8) and the numbers of newborn hippocampal neurons (**g**) (NS: *n* = 5; DZP: *n* = 4). *p < 0.05, **p < 0.01 versus NS group; ^#^*p* < 0.05, ^##^*p* < 0.01 versus SCH group; △*p* < 0.05 versus Rac group. EdU 5-ethynyl-2′-deoxyuridine.

Followingly, we performed separate experiments to explore mechanisms of MOD for behavioral improvements in OVX animals. First, we investigated whether D_1_ and D_2_ receptors contributed to the positive functions of MOD treatment in OVX mice. In the MWT, the OVX mice treated with MOD in the presence of saline showed similar spatial memory performance with the OVX mice pretreated with SCH, Rac, and combined use of SCH and Rac, respectively, before administration of MOD. The landing rates and escape latencies locating the invisible platform showed no significant variations among the different groups during the seven training days (Fig. 5a, b). Unlikely, pretreatment of Rac and combined use of SCH and Rac significantly increased the immobility time of MOD-treated OVX mice in the FST [*F*(3, 47) = 9.698, *p* < 0.0001] (Fig. 5c). Similarly, the enhanced-neurogenesis caused by MOD was decreased in the presence of SCH but increased when Rac was present [*F*(3, 10) = 17.15, *p* = 0.0003]. No significant change in the increases in newborn neurons when SCH was co-administered with Rac (Fig. 5d, e). Then, we investigated the influences of GABA_A_ receptor agonists on behavior-improving functions of MOD in OVX mice. We found that co-administration of DZP with MOD significantly prolonged the immobility time in the FST [*T*(13) = 2.378, *p* = 0.0334] (Fig. 5f) and remarkably decreased the hippocampal newborn cells of OVX mice [*T*(7) = 12.38, *p* < 0.0001] (Fig. 5e, g).

These results indicate that activation of D_1_ and D_2_ receptors contributed to the anti-depressive effects, whereas, activation of D_1_ and D_2_ receptors play opposite roles in the production of new neurons in OVX mice. Furthermore, blockade of GABA_A_ receptors also contributed to the anti-depressant effects and enhancement of hippocampal neurogenesis exerted by MOD therapy in OVX mice.

### Effects of MOD on sleep-wake behavior in OVX mice

**Fig. 6:**
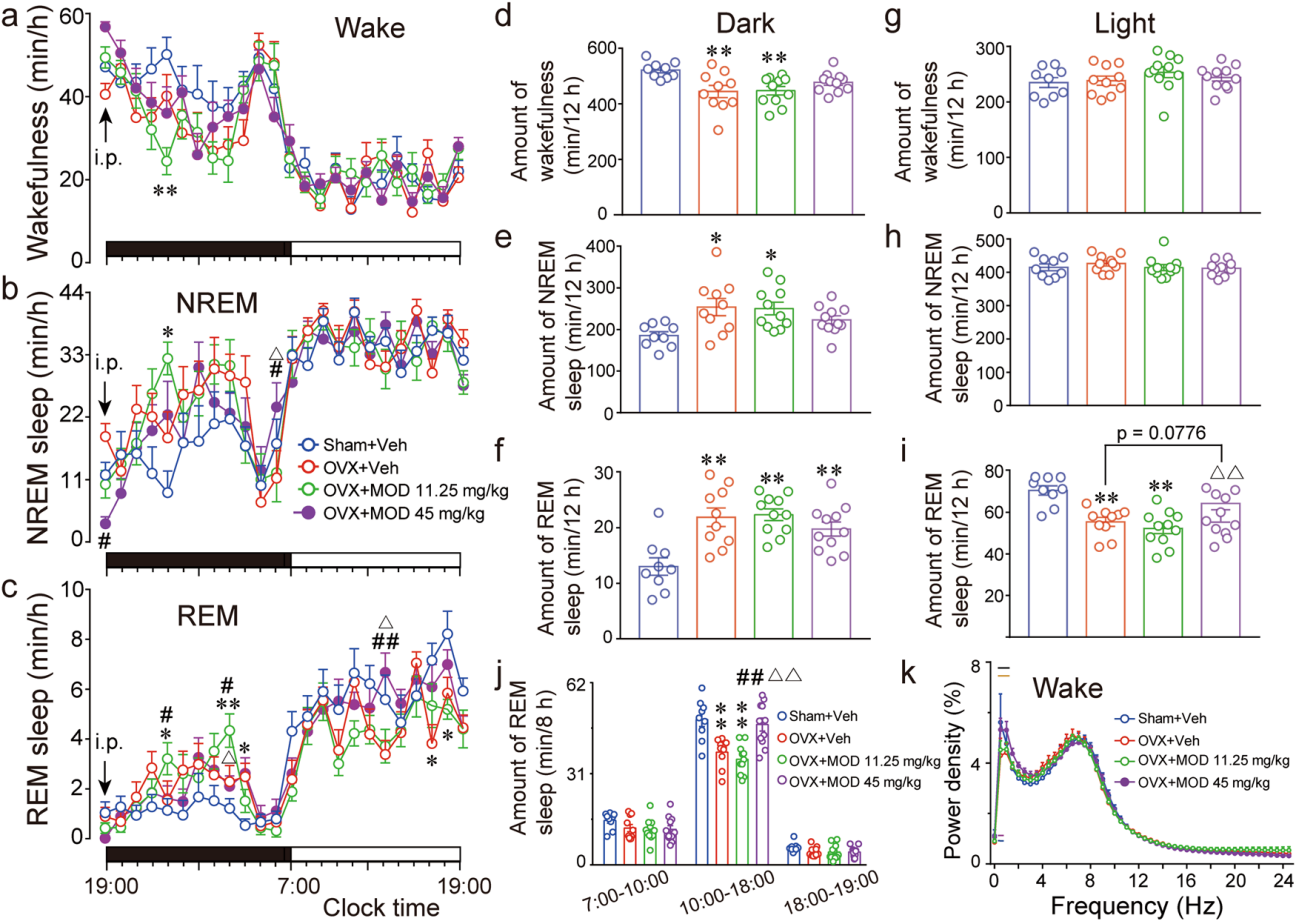
Modafinil ameliorates ovariectomy-induced decreases in daytime REM sleep and impairments of delta electroencephalogram power of wakefulness during the active phase. **a**–**c** Time courses of average hourly wakefulness (**a**), NREM sleep (**b**), and REM sleep (**c**) during the 24 h after 8 days of modafinil (MOD) or vehicle (Veh) intraperitoneal injections in mice with the protocol for sleep recording shown in Fig. 3a. The horizontal filled and open bars on the *x*-axes indicate the 12-h active and inactive phase, respectively. **d**–**i** The total amount of wakefulness (**d**, **g**), NREM sleep (**e**, **h**), and REM sleep (**f**, **i**) during the 12-h active (19:00–07:00) (**d**–**f**) and 12-h inactive (7:00–19:00) (**g**–**i**) phase, respectively, after seven days of MOD treatment. **j** In the inactive phase, amount of REM sleep during the 3-h (7:00–10:00), 8-h (10:00–18:00), and 1-h (18:00–19:00) periods. *p < 0.05, **p < 0.01 versus Sham+Veh; ^#^*p* < 0.05, ^##^*p* < 0.01 versus OVX+Veh group; △*p* < 0.05, △△*p* < 0.01 compared with OVX+MOD 11.25 mg/kg group. Sham+Veh: *n* = 9; OVX+Veh: *n* = 10; OVX+MOD 11.25 mg/kg, OVX+MOD 45 mg/kg: *n* = 11 per group. **k** The average electroencephalogram power density of wakefulness during the 12-h active phase in different groups of mice after eight consecutive days of MOD treatment. The horizontal bars indicate statistical differences (*p* < 0.05) between groups assessed by Tukey comparisons. Under the curves: blue bars for OVX+Veh group versus Sham+Veh; purple bars for OVX+MOD 11.25 mg/kg versus Sham+Veh. Above the curves: yellow bars for OVX+MOD 45 mg/kg versus OVX+Veh group; black bars for OVX+MOD 45 mg/kg versus OVX+MOD 11.25 mg/kg group. Sham+Veh: *n* = 9; OVX+Veh, OVX+MOD 11.25 mg/kg, OVX+MOD 45 mg/kg: *n* = 10 per group.

Since sleep disruption is a common symptom of menopausal women and has tremendous impacts on emotion, cognition, and brain plasticity, we further examined whether sleep/wake behavior differed in OVX mice receiving either MOD or vehicle treatments. EEGs were recorded and analyzed with the protocol shown in Fig. 3a. During the active phase from 19:00 to 7:00, two-way repeated ANOVAs revealed significant differences among the different groups in terms of hourly wakefulness [*F*(3, 37) = 5.092, *p* = 0.0047], NREM sleep [*F*(3, 37) = 4.393, *p* = 0.0097], and REM sleep [*F*(3, 37) = 8.885, *p* = 0.0001] (Fig. 6a–c). Compared with ovary-intact mice, vehicle-treated OVX mice had a decrease in wakefulness and an increase in NREM and REM sleep, which could not be significantly improved by chronic MOD administration (Fig. 6a–f). Then we analyzed the EEG power spectrum of mice in different groups. Compared with sham mice, OVX mice displayed a significant decrease in the EEG power of wakefulness at frequencies of 0.5 and 1 Hz, which were significantly increased by chronic MOD treatment at 45 mg/kg [*F*(3, 35) = 31.11, *p* < 0.0001] (Fig. 6k).

During the inactive phase from 7:00 to 19:00, OVX mice showed no significant differences in the amount of wakefulness (Fig. 6a, g) or NREM sleep (Fig. 6b, h) compared with sham mice. However, OVX mice showed a remarkable decrease in the amount of REM sleep [*F*(3, 37) = 10.37, *p* < 0.0001, Fig. 6c, i], which was dose-dependently and significantly ameliorated by chronic MOD treatment during the 8-h inactive phase from 10:00 to 18:00 [*F*(3, 37) = 12.28, *p* < 0.0001, Fig. 6j]. Less REM sleep episodes with a longer duration between 2 and 4 min [F(3, 37) = 5.324, *p* = 0.0038] may result in less REM sleep in OVX mice. When MOD was administered for eight consecutive days, the OVX mice had more REM sleep episodes [*F*(3, 37) = 5.615, *p* = 0.0028] and more NREM-to-REM transitions [*F*(3, 37) = 8.84, *p* = 0.0002] than vehicle-treated OVX mice (Fig. S2).

## Discussion

This is to our knowledge the first study to address the use of MOD in menopausal animals. The most intriguing findings of the present study in terms of the effects of MOD in OVX mice include anti-depressant-like activity and promotion of spatial memory performance, as well as some effects on the aberrant sleep-wake behavior. The underlying mechanisms for the restoration of emotional or cognitive behaviors by MOD treatment may involve increasing synaptic plasticity and neurogenesis in the hippocampus. In addition, previous studies on the effects of MOD have usually been performed shortly after drug administration and have used relatively high doses^12^. Conversely, here, we found that MOD administration during the active phase of the animals at a low-dose equivalent to clinical use in humans^39^ restores behavioral performance.

In the current study, we found that eight-day MOD administration at a higher dose significantly ameliorated OVX-induced deficits in the acquisition abilities for spatial memory but did not significantly ameliorate the spatial memory retrieval impairments. Similar results were previously found in normal rats that underwent chronic treatment with MOD. Of note, the lower dose of MOD (11.25 mg/kg) impaired spatial performance compared with the sham group, which might reflect the locomotor disabilities rather than cognitive deficits. Unlikely, Tsanov et al. showed no locomotor change with repeated administration of MOD at 10 or 64 mg/kg for normal rats in the WMT^40^. Animal strain, time of drug administration, time of spatial performance assessment, and normal or pathological animals may account for the differences in the findings.

Neural plasticity refers to the brain’s ability to change its structure and function during environmental challenges or pathological events. It involves multiple processes at the molecular and cellular levels and involves the regulation of neurogenesis and synaptic transmission. LTP, characterized by a sustained enhancement of excitatory synaptic efficacy, is widely believed to be one of the neural mechanisms of memory formation but also mood regulation^41,42^. In patients subjected to depression, LTP in the hippocampus was observed to be attenuated. Depression also downregulates synaptic proteins and growth factors required for hippocampal LTP^43^. The antidepressant drugs ketamine and fluoxetine attenuate the memory impairments and increase the amplitude of hippocampal LTP in depressive-like mice^44^. It has been proved that new neurons in the adult DG of rodents synaptically link into hippocampal circuits and involve in functional integration^45,46^. A lack of adult neurogenesis has been observed to inhibit hippocampus-dependent memory formation^47^. Findings have also revealed a direct role for adult neurogenesis in limiting depressive-like behavior. For instance, mice lacking newly born neurons in the DG area immobilized more quickly in the FST^48^. Our studies revealed that MOD treatment in OVX mice prevented the decline of LTP maintenance and neurogenesis, suggesting that the pro-cognitive and anti-depressant actions of MOD may be partially due to the promotion of hippocampal plasticity.

Several rodent studies have shown that ovarian hormones exert powerful suppression of sleep during the active phase when rats are normally awake. However, an E_2_ supplement significantly increased REM sleep during the inactive phase, when the recovery period starting at lights-on following 6-h sleep deprivation in OVX rats^49–51^. These results suggest that animals with removed ovaries might have less wakefulness in the active phase and decreased REM sleep in the inactive phase. Similar sleep disturbances in a time-of-day-dependent manner have been observed in OVX mice in this study. During the active period, in addition to having less arousal time, the OVX mice have reduced spectral power at delta waves (DW, generally 0.5-1 Hz), which was significantly ameliorated by chronic MOD treatment. During wakefulness, when LTP is induced in the motor cortex, a large and enduring increase of delta power over the ipsilateral frontal cortex and an increase of brain excitability have been clearly observed^52,53^. These findings suggest that lower EEG power of DW in the awake state may reflect daytime fatigue of menopausal women, which could be reversed by MOD. Hence, the DW may provide an objective indicator of vigilance measure.

Daytime sleepiness usually reflects poor sleep at night. In this study, the OVX mice showed insomnia, mainly characterized by decreased REM sleep and abnormal architecture of REM sleep in the inactive phase, which was relieved by eight-day administration of MOD during the active phase. Our findings are consistent with Morgan et al.^54^, who found that oral MOD taken daily in the morning for three consecutive weeks normalized nocturnal sleep architecture with an increase in REM sleep time and a decrease in REM sleep onset latencies in abstinent cocaine users. Recent studies revealed a causal link between REM sleep and hippocampus-dependent memory^55,56^, and a crucial role of REM sleep for relieving negative mood^57,58^. Meanwhile, the molecular processes of synaptic plasticity in the hippocampus are strongly activated during REM sleep^56,59^. As a result, the promotion of REM sleep by MOD therapy during the inactive phase may help to exert behavioral and plasticity improvements in OVX mice.

Although great efforts have been made in previous studies, the brain targets and mechanisms of actions of MOD are still not identified. Studies have proposed that MOD influences several neurotransmitter pathways in the brain, including dopamine, norepinephrine, orexin, histamine, glutamate, serotonin, and GABA^39,60^. In the present study, we found that both the dopaminergic and GABAergic systems are involved in MOD’s positive effects in OVX mice. Activation of GABA_A_ receptors could remarkably reverse MOD-induced anti-depressant effects and the boost of neurogenesis in OVX mice, whereas, D_1_ and D_2_ receptors play varied roles in different MOD’s actions, indicating the multi-targets of MOD in the brain. Studies have shown that estrogen facilitates effects on glutamatergic and dopaminergic neurotransmission and suppresses GABAergic inhibitory inputs^61,62^. Moreover, excitatory synaptic impairments were observed in OVX rats, in which lower spine densities in the pyramidal neurons and synaptic markers were significantly reduced in the hippocampus^9^. It is reported that GABA transporter 1-knockout mice that had excessive extracellular GABA displayed depression-like behaviors^63^. On the contrary, MOD has been shown to bind with dopamine transporters, thus increasing synaptic dopamine levels and exciting dopamine receptors^64^. Following MOD administration, hippocampal excitatory glutamatergic neurotransmission increased and GABAergic neurotransmission decreased in rats^65^. Based on previous reports together with our findings in the present study, we hypothesis that MOD may play positive roles in ovarian deficiency animals through direct activating the brain dopaminergic system or through reducing local GABAergic inhibitory tone and thus disinhibiting the transmission of excitatory neurons. However, a single existing mechanism likely cannot explain the multiple functions of MOD in OVX mice.

## Conclusions

In the present study, we report that clinically relevant doses of MOD ameliorate OVX-induced deficits in mood, memory, hippocampal plasticity, and sleep in OVX mice. Further studies are required to determine whether causal links can be found among these correlated measurements. Of course, these different domains may also work in concert in the brain, allowing for MOD-produced multidimensional functions. The overall findings of the present study suggest that MOD could be addressed as a promising therapeutic candidate for women in menopause.

## Supplementary information

Supplementary figure legends

Mice treated with either modafinil or vehicle after ovariectomies do not show any anxiety-like behaviors in open-field and elevated plus-maze tests

Modafinil improves ovariectomy-induced aberrant REM-sleep architecture during the inactive phase
