# Supplementary material for: Chronic modafinil therapy ameliorates depressive-like behavior, spatial memory and hippocampal plasticity impairments, and sleep-wake changes in a surgical mouse model of menopause
Source: Transl Psychiatry. 2021 Feb 8;11:116. doi: 10.1038/s41398-021-01229-6 (PMC7870893; doi:10.1038/s41398-021-01229-6)
Supplement: Supplementary file 1 — Supplementary figure legends [file 41398_2021_1229_MOESM1_ESM.docx]

**Supplementary figure legends**

**Fig. S1 Mice treated with either modafinil or vehicle after ovariectomies do not show any anxiety-like behaviors in open-field and elevated plus-maze tests.**

(a–d) In the 10-min open field test (OFT), total distance moved (a), average speed (b), the percentage of time spent in the center (c), and the percentage of distance traveled in the center (d) were recorded and analyzed in different groups of mice. (e–h) In the 5-min elevated plus-maze test (EPT), mice were assessed for the total distance moved (e), average speed (f), the percentage of entries into open arms (g), and duration spent in the open arms (h). Sham: n = 15 (OFT) or 14 (EPT), OVX (ovariectomy): n = 14; MOD (modafinil) 11.25 mg/kg: n = 8; MOD 45 mg/kg: n = 15.

**Fig. S2 Modafinil improves ovariectomy-induced aberrant REM-sleep architecture during the inactive phase.**

(a–d) REM-sleep architecture during the 8-h inactive phase (10:00–18:00), including episode numbers (a), number of REM-sleep bouts with different duration (b), mean duration of REM sleep (c), and conversions between S (NREM sleep), W (wakefulness), and R (REM sleep) stages (d) after eight days of modafinil (MOD) treatment in mice with ovariectomies (OVXs). * *p* < 0.05, ** *p* < 0.01 versus sham; # *p* < 0.05 versus OVX; △ *p* < 0.05, △△ *p* < 0.01 versus MOD 11.25 mg/kg group. Sham: n = 9; OVX: n = 10; MOD 11.25 mg/kg, MOD 45 mg/kg: n = 11 per group.
