# Supplementary figures and images for: Chronic modafinil therapy ameliorates depressive-like behavior, spatial memory and hippocampal plasticity impairments, and sleep-wake changes in a surgical mouse model of menopause
Source: Transl Psychiatry. 2021 Feb 8;11:116. doi: 10.1038/s41398-021-01229-6 (PMC7870893; doi:10.1038/s41398-021-01229-6)

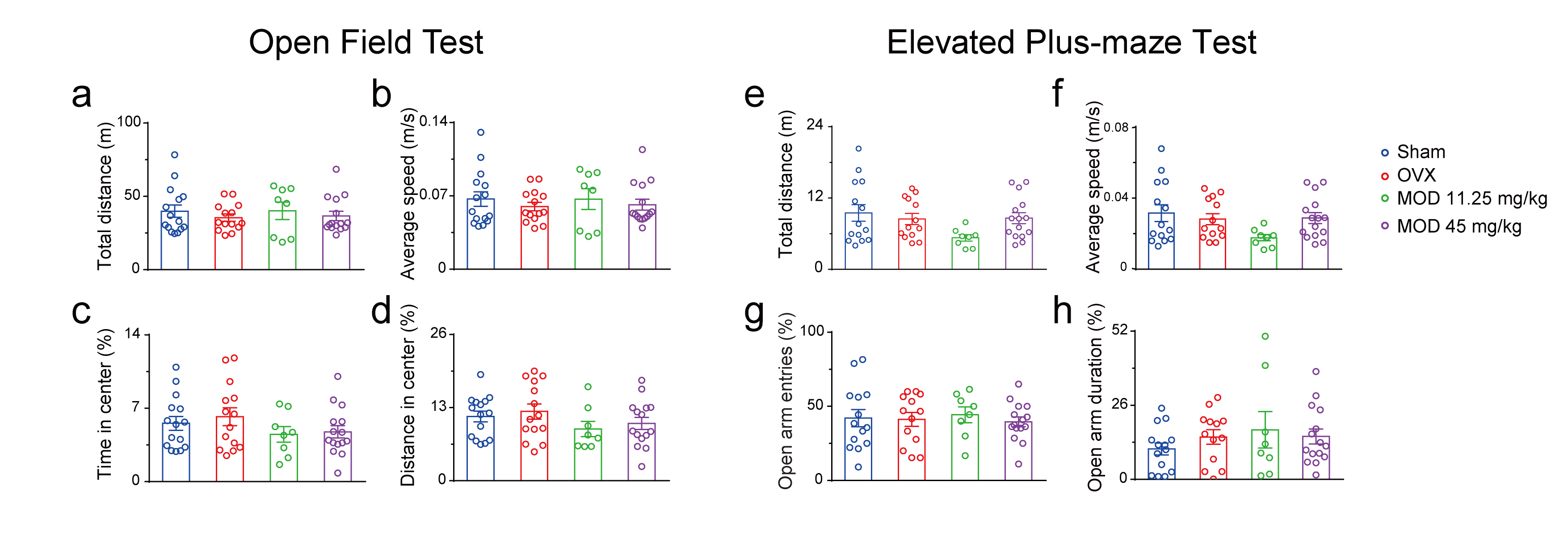

Supplement: Supplementary file 2 — Mice treated with either modafinil or vehicle after ovariectomies do not show any anxiety-like behaviors in open-field and elevated plus-maze tests [file 41398_2021_1229_MOESM2_ESM.tif]

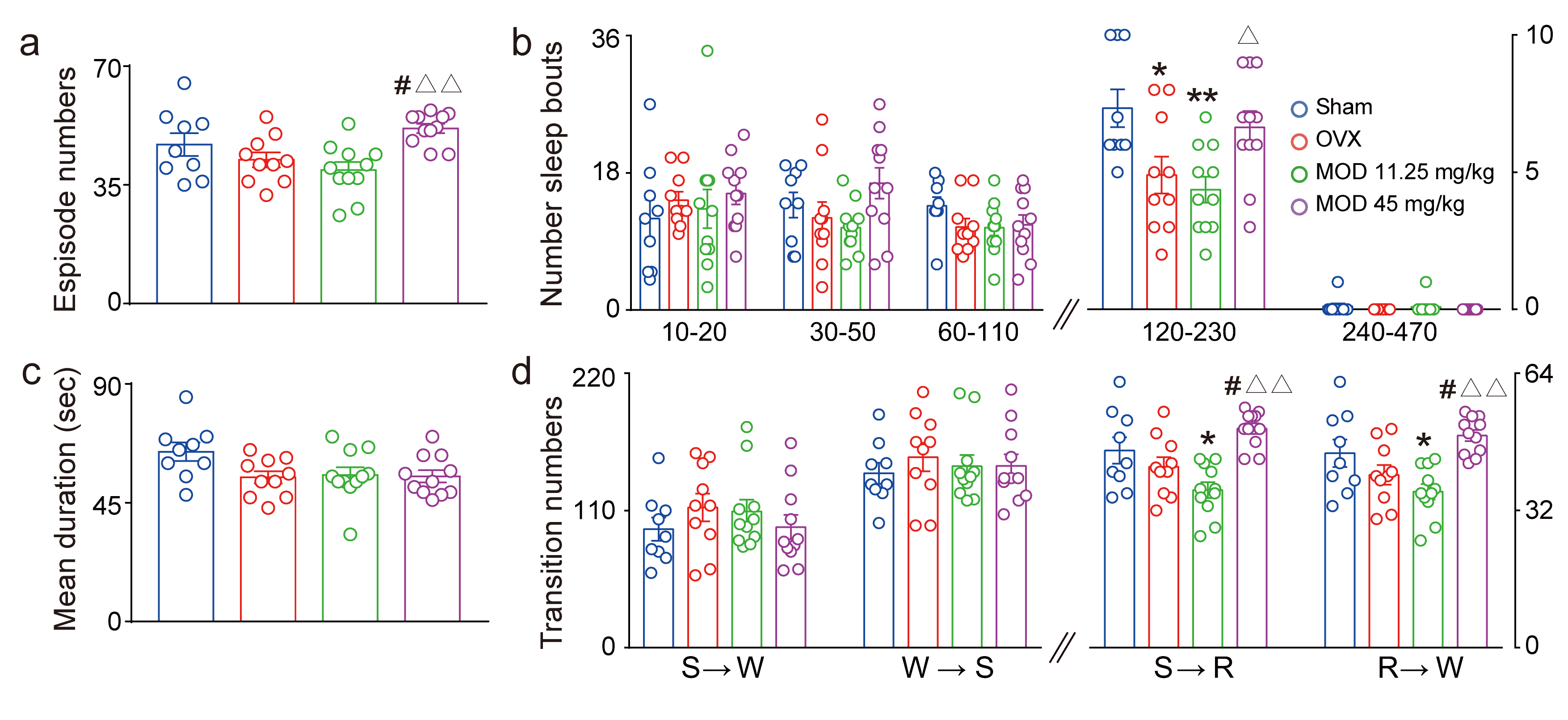

Supplement: Supplementary file 3 — Modafinil improves ovariectomy-induced aberrant REM-sleep architecture during the inactive phase [file 41398_2021_1229_MOESM3_ESM.tif]
